# In vitro and in vivo bactericidal activity of ceftazidime-avibactam against Carbapenemase–producing *Klebsiella pneumoniae*

**DOI:** 10.1186/s13756-018-0435-9

**Published:** 2018-11-21

**Authors:** Wenxia Zhang, Yan Guo, Jiayin Li, Yiyuan Zhang, Yang Yang, Dong Dong, Demei Zhu, Ping He, Fupin Hu

**Affiliations:** 1 Institute of Antibiotics, Huashan Hospital, Fudan University, 12 M. Wulumuqi Rd, Shanghai, 200040 China; 2 Department of Clinical Laboratory, Shuguang Hospital Affiliated to Shanghai University of Traditional Chinese Medicine, 528 Zhangheng Rd, Shanghai, 201203 China; 3 Department of Medical Microbiology and Immunology, Shanghai Jiao Tong University School of Medicine, Shanghai, 200025 China; 4 Key Laboratory of Clinical Pharmacology of Antibiotics, Ministry of Health, Shanghai, China

**Keywords:** *Klebsiella pneumoniae*, Carbapenemase, Ceftazidime-avibactam, Aztreonam, Time-kill curve assay

## Abstract

**Background:**

In recent years, the incidence of carbapenem-resistant *Enterobacteriaceae* (CRE) infections has increased rapidly. Since the CRE strain is usually resistant to most of antimicrobial agents, patients with this infection are often accompanied by a high mortality. Therefore, it instigates a severe challenge the clinical management of infection. In this study, we study the in vitro and in vivo bactericidal activity of ceftazidime-avibactam administrated either alone or in combination with aztreonam against KPC or NDM carbapenemase-producing *Klebsiella pneumoniae*, and explore a new clinical therapeutic regimen for infections induced by their resistant strains.

**Methods:**

The microdilution broth method was performed to analyze the minimal inhibitory concentration (MIC). The time-kill curve assay of ceftazidime-avibactam at various concentrations was conducted in 16 strains of KPC-2 and 1 strain of OXA-232 carbapenemase–producing *Klebsiella pneumoniae*. The in vitro synergistic bactericidal effect of ceftazidime-avibactam combined with aztreonam was determined by checkerboard assay on 28 strains of NDM and 2 strains of NDM coupled with KPC carbapenemase–producing *Klebsiella pneumoniae*. According to calculating grade, the drugs with synergistic bactericidal effect were selected as an inhibitory concentration index. The in vitro bactericidal tests of ceftazidime-avibactam combined with aztreonam were implemented on 12 strains among them. Effect of ceftazidime-avibactam antibiotic against KPC carbapenemase–producing *K. pneumoniae* strain Y8 Infection was performed in the mouse model.

**Results:**

The time-kill assays revealed that ceftazidime-avibactam at various concentrations of 2MIC, 4MIC and 8MIC showed significant bactericidal efficiency to the resistant bacteria strains. However, in 28 strains of NDM and 2 strains of NDM coupled with KPC carbapenemase- producing *Klebsiella pneumoniae*, only 7 strains appeared the susceptibility to ceftazidime-avibactam treatment, MIC_50_ and MIC_90_ were 64 mg/L and 256 mg/L, respectively. Antimicrobial susceptibility testing of ceftazidime-avibactam combined with aztreonam disclosed the synergism of two drugs in 90% (27/30) strains, an additive efficiency in 3.3% (1/30) strains, and irrelevant effects in 6.6% (2/30) strains. No antagonism was found. The subsequent bactericidal tests also confirmed the results mentioned above. Therapeutic efficacy of Ceftazidime-Avibactam against *K. pneumoniae* strain Y8 infection in mouse indicated 70% of infection group mice died within 4 days, and all mice in this group died within 13 days. Bacterial load testing results showed that there was no significant difference in the amount of bacteria in the blood between the infected group and the treatment group. However, the spleen and liver of treatment group mice showed lower CFU counts, as compare with infected group, indicating that ceftazidime-avibactam has a significant effect on the bacteria and led to a certain therapeutic efficacy.

**Conclusion:**

This study indicated ceftazidime-avibactam therapy occupied significant bactericidal effects against KPC-2 and OXA-232 carbapenemase-producing *Klebsiella pneumoniae*. While combined with aztreonam, the stronger synergistic bactericidal effects against NDM carbapenemase-producing *Klebsiella pneumoniae* were achieved.

## Background

Carbapenems are considered the most effective antibacterial agents against infections caused by multi-drug resistant gram-negative bacillus in clinical practice. However, with the broad use of carbapenems, and the emergence and widespread of carbapenem-resistant *Enterobacteriaceae* (CRE), in particular carbapenem-resistant *Klebsiella pneumoniae* (CR-KP), the clinical anti-infection treatment faces a drug-free dilemma [1–4]. Previous studies have shown that the most important resistant mechanism of CR-KP to carbapenems is production of carbapenemases including class A KPC carbapenemases, class B metallo-β-lactamases and class D OXA-48 family carbapenemases. Avibactam is a newly developed novel β-lactamase inhibitor in recent years and can efficiently inhibit class A and class D carbapenemases. As an inhibitor, avibactam can restore the antibacterial activity of ceftazidime to CR-KP. Nevertheless, it has less antibacterial efficiency against CR-KP with metallo-β-lactamase [5–7]. Knowing that aztreonam is stable against hydrolysis by class B metallo-β-lactamases, we hypothesized that supplement of aztreonam to the ceftazidime-avibactam would enhance the activity by “protecting” aztreonam from the “attack” of KPC type carbapenemase. This study aims to explore a new therapeutic regimen by administration of ceftazidime-avibactam alone or combined with aztreonam against carbapenemase-producing *Klebsiella pneumoniae*.

## Materials and methods

### Strains

A total of 47 non-repeated clinical strains of carbapenemase-producing *Klebsiella pneumoniae* were collected from 9 hospitals in 9 cities in China. Of these, 16 strains were *bla*_KPC-2_ positive, 1 was *bla*_OXA-232_ positive, 28 were *bla*_NDM_ positive, and 2 were *bla*_KPC2_ coupled with *bla*_NDM_ positive *Klebsiella pneumoniae*. All strains were identified by mass spectrometry and the gene type of carbapenemases was analyzed by PCR amplification using primers previously described and DNA sequencing [8–10]. *E. coli* ATCC 25922 were used as quality control strain for antimicrobial susceptibility testing. One *K. pneumoniae* clinical strain Y8 was used for the Infection in the mouse model.

### Antimicrobial susceptibility testing

The minimal inhibitory concentrations were determined by microbroth dilution according to Clinical and Laboratory Standards Institute (CLSI) guidelines [11]. The in vitro synergistic bactericidal effects of ceftazidime- avibactam combined with aztreonam against *bla*_NDM_-positive strains were determined by checkerboard assay referred to the published reports [12, 13]. The calculation and interpretation of fractional inhibitory concentration (FIC) was referred to the document standards [14]. FIC = MIC _drug A_ / MIC _drug A plus drug B_ + MIC_drug B_/ MIC _drug A plus drug B_. FIC ≤ 0.5 was considered as a synergistic effect, 0.5 < FIC ≤ 1 was considered as an additive effect, 1 < FIC ≤ 2 was considered as an irrelevant effect, and > 2 was considered as an antagonism.

### Time-kill assay

According to the minimal inhibitory concentration (MIC) of ceftazidime-avibactam to *bla*_KPC-2_ or *bla*_OXA-232_ producers, the bactericidal effects of ceftazidime-avibactam at various concentrations of 0.5MIC, 1MIC, 2MIC, 4MIC and 8MIC were studied by time-kill assay. In line with the results of antimicrobial susceptibility testing of ceftazidime-avibactam combined with aztreonam, and following the methods recommended in literatures [15, 16], 10 *Klebsiella pneumoniae* strains with *bla*_NDM_ and 2 *Klebsiella pneumoniae* strains with *bla*_NDM_ coupled with *bla*_KPC-2_ were randomly selected for synergistic bactericidal effects. The operation procedure is briefly described as follows: Mueller-hinton broth containing 1 × 10^5^ CFU/mL bacteria is mixed with single or combined antimicrobial agents incubated overnight with consecutive shacking at 35 °C in an atmospheric environment. Meanwhile, the same broth without antibiotics was served as a growth control. Broth samples were serially diluted at times of 0, 2, 4, 6, 8 and 10 h and smeared on a mueller-Hinton plate respectively. After overnight incubation at 35 °C, the colonies were counted. If the reduction of bacterial survival amount in sample treated with combined antibiotics was ≥2 log10 CFU/mL than those in the sample treated by single drug, it was considered to have a synergistic bactericidal effect.

### Effect of ceftazidime-avibactam against *K. pneumoniae* strain Y8 infection in the mouse model

Six-week-old BALB/c mice (female) were bought from Shanghai Laboratory Animal Company (SLAC), China. Animal experiments were performed in accordance with the Animal Ethics Committee of Shanghai Jiao Tong University. Groups of 10 mice were infected with 2.5 × 10^6^ CFU of strain Y8 via the intraperitoneal (ip) route. Then mice were treated with PBS (infection group) or ceftazidime-avibactam (treatment group) (0.375 mg/g of body weight in 0.1 ml PBS) by subcutaneous injection 4 h post infection and given every 8 h for 10 days. The survival rates of mice were measured at desired time point to assess the therapeutic efficacy of ceftazidime-avibactam. Survival curves were monitored for 15 days.

### Bacterial load in the blood and tissues of mice

To measure the efficacy of this drug, the bacterial load was measured in the blood and tissues of mice. Groups of 8 mice were infected with 2.5 × 10^6^ CFU of strain Y8 via the ip and then treated with PBS or ceftazidime-avibactam by subcutaneous injection. The antibiotic dosage and administration were same as the survival experiment mentioned above. At 3 days post infection (dpi), mice in treatment and infected group were euthanized, and the blood and tissues of mice were removed to determine the bacterial burden through bacterial dilution-plate method.

## Results

### Antimicrobial susceptibility testing

All 16 *Klebsiella pneumoniae* strains with *bla*_KPC-2_ were susceptible to ceftazidime-avibactam with MIC range for 4–8 mg/L. However, all of strains were resistant to ceftazidime with MIC_50_ of 32 mg/L and MIC_90_ of > 256 mg/L. The resistance rate of imipenem was 93.8% with MIC_50_ and MIC_90_ for 64 mg/L and 128 mg/L, respectively. The resistance rate of meropenem was 93.8% with MIC_50_ and MIC_90_ for 64 mg/L and 256 mg/L, respectively (Table 1). The MIC of ceftazidime-avibactam to one OXA-232 carbapenemase-producing *Klebsiella pneumoniae* was 2 mg/L. The resistance rate of ceftazidime-avibactam to 30 *bla*_NDM_ (including NDM plus KPC-2) positive *Klebsiella pneumoniae* strains was 76.7% with MIC range for 0.5~ 256 mg/L, MIC_50_ and MIC_90_ for 64 mg/L and 256 mg/L, respectively. The MIC range of aztreonam was 8~ > 256 mg/L with MIC_50_ and MIC_90_ were 128 mg/L and > 256 mg/L, respectively. Ceftazidime-avibactam combined with aztreonam showed synergistic effects to 90% (27/30) of strains with *bla*_NDM,_ 3.3% (1/30) showed additive effects and 6.6%(2/30) showed unrelated effects. No antagonism was found for ceftazidime-avibactam combined with aztreonam (Table 2).

**Table 1:** Minimal inhibitory concentration (MIC) of ceftazidime-avibactam against KPC-2 or OXA-232 carbapenemase-producing *Klebsiella pneumoniae*

Strain no.	Bacteria	β-lactamase	MIC (mg/L)				Associated β-lactamase
			CAZ-AVI	CAZ	IPM	MEM	
R16- Hefei	*K. pneumoniae*	KPC-2	8	128	64	64	CTX-M-14, SHV-11, DHA-1
R18- Hefei	*K. pneumoniae*	KPC-2	4	128	8	16	SHV-28, DHA-1
R19- Hefei	*K. pneumoniae*	KPC-2	4	128	16	16	SHV-12, DHA-1
R31- Beijing	*K. pneumoniae*	KPC-2	8	128	64	256	SHV-11, DHA-1
R35- Beijing	*K. pneumoniae*	KPC-2	8	128	128	256	SHV-11, DHA-1
R39-Fuzhou	*K. pneumoniae*	KPC-2	8	> 256	32	64	CTX-M-14, SHV-12, DHA-1
R42- Fuzhou	*K. pneumoniae*	KPC-2	8	> 256	32	64	CTX-M-14, SHV-12, DHA-1
R44- Fuzhou	*K. pneumoniae*	KPC-2	8	> 256	64	128	CTX-M-14, SHV-12, DHA-1
R46- Fuzhou	*K. pneumoniae*	KPC-2	8	> 256	64	64	SHV-12, DHA-1
R52- Fuzhou	*K. pneumoniae*	KPC-2	8	> 256	64	128	CTX-M-14, SHV-12, DHA-1
R53- Fuzhou	*K. pneumoniae*	KPC-2	8	> 256	64	128	CTX-M-14, SHV-12, DHA-1
R59- Hangzhou	*K. pneumoniae*	KPC-2	8	32	8	16	CTX-M-14, SHV-11, DHA-1
R60- Hangzhou	*K. pneumoniae*	KPC-2	4	32	4	4	CTX-M-14, SHV-11, DHA-1
JSD-Shanghai	*K. pneumoniae*	KPC-2	8	128	128	512	CTX-M-14, SHV-11, DHA-1
WJQ-Shanghai	*K. pneumoniae*	KPC-2	8	> 256	128	256	CTX-M-14, SHV-11, DHA-1
LDX-Shanghai	*K. pneumoniae*	KPC-2	8	> 256	32	64	CTX-M-55, SHV-31, DHA-1
PED-Shanghai	*K. pneumoniae*	OXA-232	2	> 32	1	4	CTX-M-15, SHV-1

Note: *CAZ-AVI* Ceftazidime-avibactam, *CAZ* Ceftazidime, *IPM* Imipenem, *MEM* Meropenem

**Table 2:** Results of MIC and antimicrobial susceptibility testing of ceftazidime-avibactam single dosing and combined with aztreonam against NDM + KPC-2 carbapenemase-producing *Klebsiella pneumoniae* in 30 strains

Strain no.	Bacteria	β-lactamase	MIC(mg/L) single dosing		MIC(mg/L) Combined dosing		FIC value	Associated β-lactamase
			ATM	CAZ-AVI	ATM	CAZ-AVI	ATM + CAZ-AVI	
R078 Anhui	*K. pneumoniae*	NDM	256	32	8	1	0.06	SHV-28, DHA-1, CTX-M-15
R080 Hainan	*K. pneumoniae*	NDM	128	4	32	0.25	0.31	SHV-11, DHA-1, CTX-M-14
R081 Hainan	*K. pneumoniae*	NDM	1024	8	64	2	0.31	SHV-11, DHA-1, CTX-M-14
R082 Hainan	*K. pneumoniae*	NDM	256	64	16	1	0.08	SHV-11, DHA-1, CTX-M-15, CTX-M-14
R083 Hainan	*K. pneumoniae*	NDM	128	64	32	0.5	0.26	SHV-12, DHA-1, CTX-M-15
R084 Hainan	*K. pneumoniae*	NDM	512	64	16	1	0.05	SHV-12, DHA-1
R085 Hainan	*K. pneumoniae*	NDM	1024	128	32	2	0.05	SHV-12, DHA-1, CTX-M-15
R086 Hainan	*K. pneumoniae*	NDM	128	64	16	0.5	0.13	SHV-12, DHA-1, CTX-M-15
R088 Hainan	*K. pneumoniae*	NDM	32	2	4	0.5	0.38	SHV-11, DHA-1, CTX-M-14
R093 Hebei	*K. pneumoniae*	NDM	32	64	4	0.5	0.13	SHV1, DHA-1, CTX-M-14
R094 Hebei	*K. pneumoniae*	NDM	32	64	8	0.25	0.25	SHV-12, DHA-1, CTX-M-14
R095 Hebei	*K. pneumoniae*	NDM	32	2	8	0.25	0.38	SHV-12, DHA-1, CTX-M-15
R096 Henan	*K. pneumoniae*	NDM	16	64	16	0.5	1.01	SHV1, DHA-1, CTX-M-15
R097 Henan	*K. pneumoniae*	NDM	8	256	4	128	1.00	SHV1, DHA-1, CTX-M-14
R098 Henan	*K. pneumoniae*	NDM	256	64	32	1	0.14	DHA-1
R100 Shanxi	*K. pneumoniae*	NDM	8	32	4	32	1.50	SHV-78, DHA-1, CTX-M-14
R101 Shanxi	*K. pneumoniae*	NDM	256	128	16	1	0.07	SHV-78, DHA-1, CTX-M-14
R102 Shanxi	*K. pneumoniae*	NDM	32	256	4	1	0.13	SHV-78, DHA-1, CTX-M-14
R103 Shanxi	*K. pneumoniae*	NDM	128	64	32	0.25	0.25	SHV1, DHA-1, CTX-M-15
R106 Sanxi	*K. pneumoniae*	NDM	128	64	8	1	0.08	SHV-12, DHA-1
R110 Sanxi	*K. pneumoniae*	NDM	128	256	8	1	0.07	SHV-12, DHA-1
R113 Sanxi	*K. pneumoniae*	NDM	128	64	16	0.5	0.13	SHV-12, DHA-1
R122 Tianjin	*K. pneumoniae*	NDM	32	1	4	0.25	0.38	SHV-12, DHA-1, CTX-M-14
R126 Tianjin	*K. pneumoniae*	NDM	512	64	16	1	0.05	SHV-12, DHA-1, CTX-M-14
R127 Tianjin	*K. pneumoniae*	NDM	256	128	32	1	0.13	SHV2, DHA-1
R128 Zhejiang	*K. pneumoniae*	NDM	128	256	8	2	0.07	SHV1, DHA-1
R129 Zhejiang	*K. pneumoniae*	NDM	4	0.5	0.5	0.125	0.38	SHV-12, DHA-1, CTX-M-15
R136 Zhejiang	*K. pneumoniae*	NDM	256	64	16	1	0.08	SHV-12, DHA-1
R148 Tianjing	*K. pneumoniae*	KPC-2,NDM	2048	8	256	2	0.38	SHV-12, DHA-1, CTX-M-14
R153 Henan	*K. pneumoniae*	KPC-2,NDM	2048	128	128	8	0.13	SHV-12, DHA-1

### Time-kill assay

**Fig. 1:**
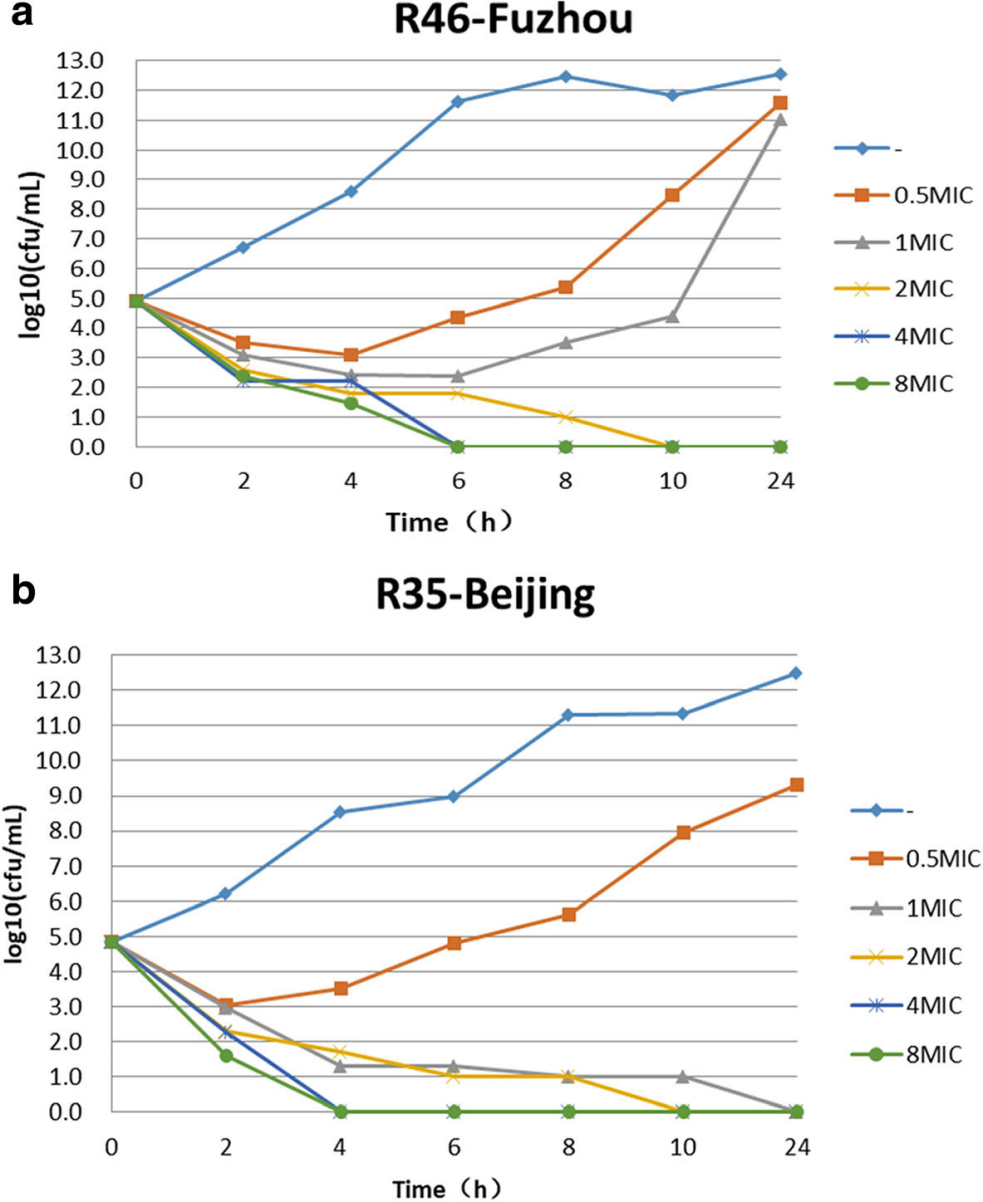
Bactericidal curve plots of ceftazidime-avibactam at various concentrations against KPC-2 carbapenemase-producing *Klebsiella pneumoniae*

**Fig. 2:**
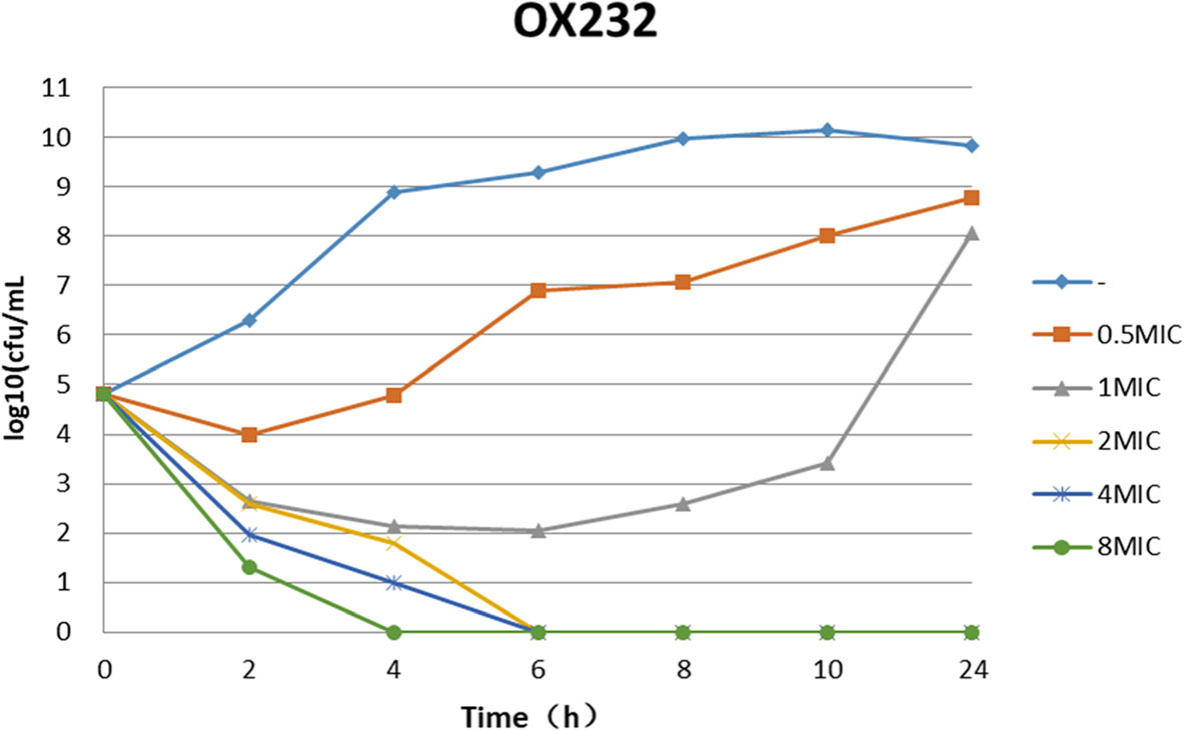
Bactericidal curve plots of ceftazidime-avibactam at various concentrations against OXA-232 carbapenemase-producing *Klebsiella pneumoniae*

**Fig. 3:**
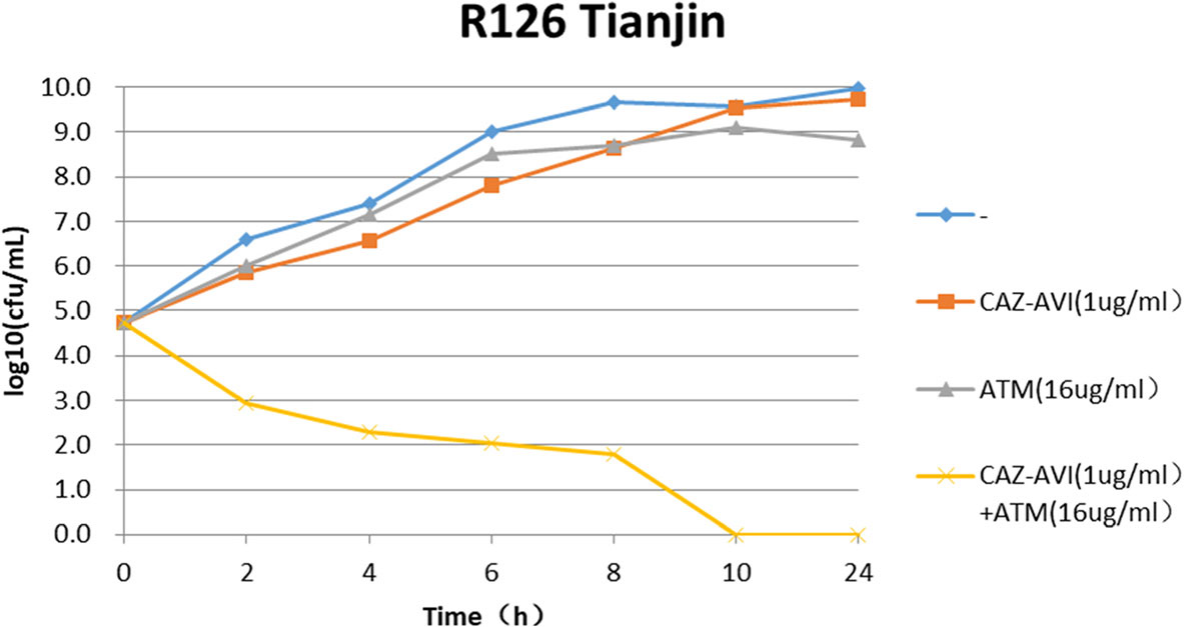
Bactericidal curve plots of ceftazidime-avibactam combined with aztreonam against NDM carbapenemase-producing *Klebsiella pneumoniae*

The results of time-kill assays showed that all *bla*_KPC-2_ or *bla*_OXA-232_ positive *K. pneumoniae* strains rebounded to grow 4 to 6 h at 0.5 MIC of ceftazidime-avibactam. At the concentration of 1 MIC ceftazidime-avibactam, 23.5% (4/17) of strains declined stably 2 h after dosing and no colonies were detected at 24 h, however, 76.5% (13/17) of strains rebounded to grow in 4–6 h. At the concentrations of 2MIC, 4MIC or 8MIC of ceftazidime-avibactam, it showed a significant bactericidal effectiveness for either *bla*_KPC_ or *bla*_OXA-232_ positive *K. pneumoniae* and the colony growth was undetected after 24 h incubation for most of strains (Fig. 1 and Fig. 2). For *bla*_NDM_ positive *K. pneumoniae*, ceftazidime-avibactam combined with aztreonam showed a significant bactericidal effectiveness and the colony growth was undetected after 10 h incubation (Fig. 3).

### Therapeutic efficacy of ceftazidime-avibactam against *K. pneumoniae* strain Y8 infection in mouse

**Fig. 4:**
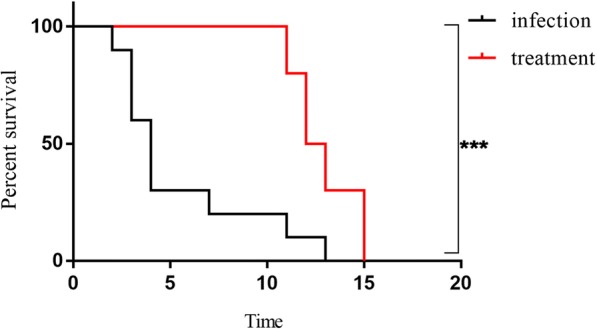
Therapeutic efficacy of ceftazidime-avibactam against *K. pneumoniae* strain Y8 infection in mouse. Mice were infected with 2.5 × 10^6^ CFU of strain Y8 via the ip route and then treated with PBS or ceftazidime-avibactam by subcutaneous injection. Their survival was assessed daily for 15 days (*n* = 10). ∗*P* < 0.05; ∗∗∗*P* < 0.001

Mice were infected with 2.5 × 10^6^ CFU of strain Y8 and treated with PBS or ceftazidime-avibactam for 10 days. 70% of infection group mice died within 4 days, and all mice in this group died within 13 days (Fig. 4). All treatment group mice survived at 10 dpi with the antibiotic applied every 8 h, whereas 100% of mice in this group died within 4 days after the antibiotic treatment stopped (Fig. 4).

### Bacterial load in the blood and tissues of mice

**Fig. 5:**
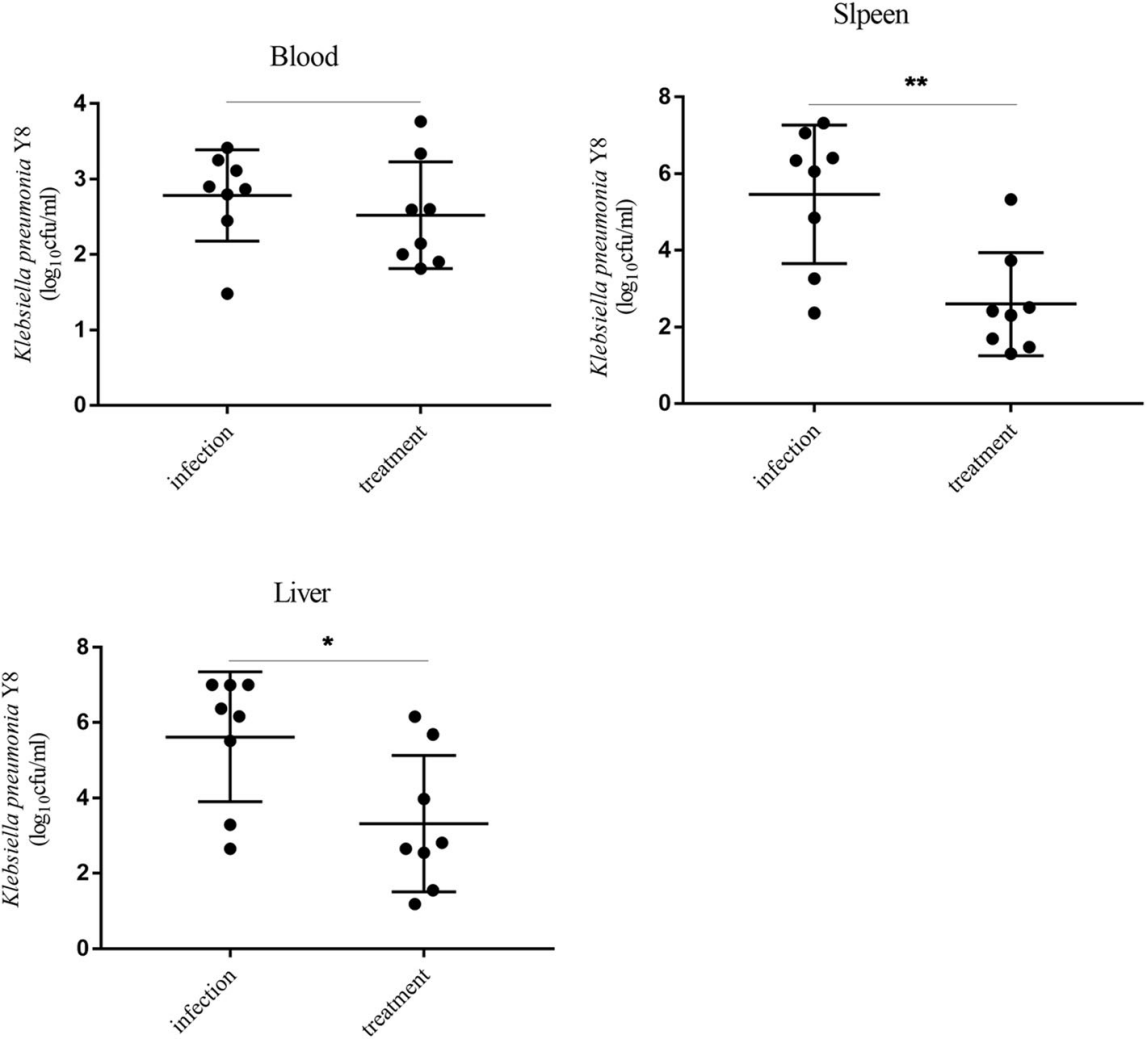
Bacterial burden in blood, spleen and liver of mice at 3 days post infection (dpi). Mice were infected with 2.5 × 10^6^ CFU of strain Y8 via the ip route and then treated with PBS or ceftazidime-avibactam by subcutaneous injection. At 3dpi, the viable bacteria of blood, spleen and liver was determined by plating serial dilutions on agar plates (*n* = 8). ∗*P* < 0.05; ∗∗*P* < 0.01

Mice were infected with 2.5 × 10^6^ CFU of strain Y8 via the ip and then treated with PBS or ceftazidime-avibactam. The viable bacteria were quantified in blood, liver and spleen at 3 dpi. The results showed that there was no significant difference in the amount of bacteria in the blood between the infected group and the treatment group. However, the spleen and liver of treatment group mice showed lower CFU counts, as compare with that of infected group, indicating that the antibiotic has significant effect on the bacteria and ceftazidime-avibactam led to a certain therapeutic efficacy (Fig. 5).

## Discussion

In the recent decade, the prevalence and dissemination of CR-KP has posed a serious challenge in healthcare facilities in the world. The data from the Centers for Disease Control, USA disclosed the infection incidence of CR-KP increased from 1.2% in 2001 to 4.2% in 2011. In annual of 140,000 cases with *Enterobacteriaceae* infections, 9300 cases (6.6%) were infected by these multidrug-resistant bacteria [17]. According to CHINET surveillance data, the resistance rate of *Klebsiella pneumoniae* to carbapenems were significantly increasing from 3% in 2005 to 20% in 2017 [18]. Due to lack of effective antibacterial agents, infections due to CR-KP especially for hypervirulent *Klebsiella pneumoniae* usually accompany with high mortality [19, 20]_._ Carbapenemases are the major resistance mechanism of *Klebsiella pneumoniae* to carbapenems. Currently, the common carbapenemase among *K. pneumoniae* clinical strains include Ambler class A, Ambler class B and Ambler class D (eg. *bla*_OXA-48_, and *bla*_OXA-232_). *bla*_KPC-2_ is the most common carbapenemase in class A enzymes which can hydrolyze almost all of β-lactam antibiotics. *bla*_NDM_ is the most common carbapenemase in class B metallo-β-lactamase which can hydrolyze all β-lactam antibiotics except aztreonam. Previous studies have shown the CR-KP strains isolated from children in China mainly produce *bla*_NDM_ [21]. *bla*_OXA-48_ carbapenemase are predominantly existent in *Klebsiella pneumoniae* isolated in Tokyo and Europe [22]. In China, *bla*_OXA-181_ and *bla*_OXA-232_ have also been detected among *Klebsiella pneumoniae* clinical strains [23, 24]_._

Since CRE are usually extensively drug resistant, infections due to CRE are often associated with a high mortality. Therefore, a serious challenge of anti-infection therapy for CRE has been raised in clinical practice. Studies have revealed that compared to use carbapenems antibiotics alone, in the combination of carbapenems with other antibacterial agents such as tigecycline or polymyxin [25–28], amikacin [29] or fosfomycin [30] can considerably improve the outcomes of patient with CR-KP infection. Laurent adopted a combined pharmacotherapy regimen of dual carbapenems to provide a new approach in the treatment of CRE-induced infections [31]. As a novel β-lactam/β-lactamase inhibitor, studies have shown that ceftazidime-avibactam can successfully cure the infections due to *Enterobacteriaceae* with *bla*_KPC_ [32–35]. Nevertheless, it is worth to be noticed that with the increasing application of ceftazidime-avibactam, the resistant strains and failed cases have been reported [6, 7, 36]. Our results showed that all of 16 *bla*_KPC-2_ positive and 1 of *bla*_OXA-232_ positive *Klebsiella pneumoniae* were susceptible to ceftazidime-avibactam with MIC_50_ and MIC_90_ for both 8 mg/L. Time-kill assays demonstrated that it showed a significant bactericidal effectiveness for either *bla*_KPC_ or *bla*_OXA-232_ positive *K. pneumoniae* at the concentrations of 2MIC, 4MIC or 8MIC of ceftazidime-avibactam. 1 and Fig. 2). At 1 MIC of ceftazidime-avibactam, most of *K. pneumoniae* strains started to regrowth within 4–6 h. These results mean that the dosage of ceftazidime-avibactam is very important in the treatment of infections caused by *bla*_KPC_ or *bla*_OXA-232_ positive *K. pneumoniae* [37].

In this study, 76.7% of *bla*_NDM_ positive *Klebsiella pneumoniae* were resistance to ceftazidime-avibactam with MIC_50_ and MIC_90_ for 64 mg/L and 256 mg/L, respectively. Because avibactam can not inhibit the activity of metallo-β-lactamase, ceftazidime-avibactam monotherapy was ineffective for infections caused by *bla*_NDM-1_ positive *Klebsiella pneumoniae* [38]. According to the results of time-kill assay, ceftazidime-avibactam combined with aztreonam is necessary for the treatment of infection due to *bla*_NDM_ positive *K. pneumoniae* based on the characteristics of weak hydrolysis capacity of metallo-β-lactamases to aztreonam. Eric Wenzler et al. also demonstrated that the combination of ceftazidime-avibactam and aztreonam had a synergistic bactericidal effect against class B metallo-β-lactamase-producing gram-negative bacteria [39]. Simultaneously, Benjamin Davido et al. reported that two cases infected either with metallo-β-lactamase-producing *Klebsiella pneumoniae* or *Pseudomonas aeruginosa* were successfully cured by combined administration of ceftazidime-avibactam and aztreonam [40]. The results of antimicrobial susceptibility testing indicated 90% (27/30) strains showed a synergistic effect for ceftazidime-avibactam combined with aztreonam. After combined with aztreonam, the MICs of ceftazidime-avibactam for 27 strains were reduced 4–256 times than ceftazidime-avibactam alone and all of them were susceptible to ceftazidime-avibactam with MIC ≤8 mg/L. Subsequently, the bactericidal curve tests performed on 12 strains of *Klebsiella pneumoniae* with a synergistic effect were also shown the consistent synergistic bactericidal effects. The reduction of bacterial colonies number was >2 log_10_ CFU/ mL compared with monotherapy and no colonies were detected after 24 h. In addition to strain of R148 showing the synergism 8 h after combined therapy, three *bla*_NDM_ positive *Klebsiella pneumoniae* clinical strains of R96, R97 and R100 did not demonstrate the synergistic effects after combined use with aztreonam, implying the other mechanisms of drug resistance may exist and require the further investigation in the future.

## Conclusions

In summary, as a compound preparation of novel enzyme inhibitor, ceftazidime-avibactam possesses visible advantages in the treatment of class A and class D type carbapenemase–producing *Klebsiella pneumoniae* clinical isolates. In addition, if combined with aztreonam, it can also play a synergetic bactericidal effects against infections caused by *bla*_NDM_ positive *Klebsiella pneumoniae* clinical strains.

## Abbreviations

CAZ: Ceftazidime
CAZ-AVI: Ceftazidime-avibactam
CRE: Carbapenem-resistant *Enterobacteriaceae*
CR-KP: Carbapenem-resistant *Klebsiella pneumoniae*
FIC: Fractional inhibitory concentration
IPM: Imipenem
MEM: Meropenem
MIC: Minimal inhibitory concentration

## References

[1] Ducomble T, Faucheux S, Helbig U, Kaisers UX, Konig B, Knaust A, Lubbert C, Moller I, Rodloff AC, Schweickert B, Eckmanns T (2015). Large hospital outbreak of KPC-2-producing *Klebsiella pneumoniae*: investigating mortality and the impact of screening for KPC-2 with polymerase chain reaction. J Hosp Infect.

[2] Kim JO, Song SA, Yoon EJ, Shin JH, Lee H, Jeong SH, Lee K (2017). Outbreak of KPC-2-producing *Enterobacteriaceae* caused by clonal dissemination of *Klebsiella pneumoniae* ST307 carrying an IncX3-type plasmid harboring a truncated Tn4401a. Diagn Microbiol Infect Dis.

[3] Yang J, Ye L, Guo L, Zhao Q, Chen R, Luo Y, Chen Y, Tian S, Zhao J, Shen D, Han L (2013). A nosocomial outbreak of KPC-2-producing *Klebsiella pneumoniae* in a Chinese hospital: dissemination of ST11 and emergence of ST37, ST392 and ST395. Clin Microbiol Infect.

[4] Liu J, Yu J, Chen F, Yu J, Simner P, Tamma P, Liu Y, Shen L (2018). Emergence and establishment of KPC-2-producing ST11 *Klebsiella pneumoniae* in a general hospital in Shanghai, China. Eur J Clin Microbiol Infect Dis.

[5] Zasowski EJ, Rybak JM, Rybak MJ (2015). The beta-lactams strike Back: ceftazidime-avibactam. Pharmacotherapy.

[6] Shields Ryan K., Nguyen M. Hong, Chen Liang, Press Ellen G., Kreiswirth Barry N., Clancy Cornelius J. (2018). Pneumonia and Renal Replacement Therapy Are Risk Factors for Ceftazidime-Avibactam Treatment Failures and Resistance among Patients with Carbapenem-Resistant Enterobacteriaceae Infections. Antimicrobial Agents and Chemotherapy.

[7] Shields RK, Chen L, Cheng S, Chavda KD, Press EG, Snyder A, Pandey R, Doi Y, Kreiswirth BN, Nguyen MH, Clancy CJ (2017). Emergence of ceftazidime-avibactam resistance due to plasmid-borne blaKPC-3 mutations during treatment of Carbapenem-resistant *Klebsiella pneumoniae* infections. Antimicrob Agents Chemother.

[8] Zhu J, Sun L, Ding B, Yang Y, Xu X, Liu W, Zhu D, Yang F, Zhang H, Hu F (2016). Outbreak of NDM-1-producing *Klebsiella pneumoniae* ST76 and ST37 isolates in neonates. Eur J Clin Microbiol Infect Dis.

[9] Woodford N, Fagan EJ, Ellington MJ (2006). Multiplex PCR for rapid detection of genes encoding CTX-M extended-spectrum (beta)-lactamases. J Antimicrob Chemother.

[10] Poirel L, Walsh TR, Cuvillier V, Nordmann P (2011). Multiplex PCR for detection of acquired carbapenemase genes. Diagn Microbiol Infect Dis.

[11] Clinical and Laboratory Standards Institute. Performance Standards for Antimicrobial Susceptibility Testing[S]: Twenty-seventh Informational Supplement. PA: CLSI; 2017.

[12] Bercot B, Poirel L, Dortet L, Nordmann P (2011). In vitro evaluation of antibiotic synergy for NDM-1-producing *Enterobacteriaceae*. J Antimicrob Chemother.

[13] Elemam A, Rahimian J, Doymaz M (2010). In vitro evaluation of antibiotic synergy for polymyxin B-resistant carbapenemase-producing *Klebsiella pneumoniae*. J Clin Microbiol.

[14] Gunderson BW, Ibrahim KH, Hovde LB, Fromm TL, Reed MD, Rotschafer JC (2003). Synergistic activity of colistin and ceftazidime against multiantibiotic-resistant Pseudomonas aeruginosa in an in vitro pharmacodynamic model. Antimicrob Agents Chemother.

[15] Paevskii SA. A means for determining the bactericidal activity of the tissues during the treatment of orthopedic patients by transosseous osteosynthesis methods. Klin Lab Diagn. 1993:25–9. 7994538

[16] Norden CW, Wentzel H, Keleti E (1979). Comparison of techniques for measurement of in vitro antibiotic synergism. J Infect Dis.

[17] US Centers for Disease Control and Prevention. Antibiotic Resistance Theats in the United States. 2013. https://www.cdc.gov/drugresistance/threat-report-2013/pdf/ar-threats-2013-508.pdf.

[18] Hu FP, Guo Y, Zhu DM, Wang F, Jiang XF, Xu YC, Zhang XJ, Zhang CX, Ji P, Xie Y, Kang M, Wang CQ, Wang AM, Xu YH, Shen JL, Sun ZY, Chen ZJ, Ni YX, Sun JY, Chu YZ, Tian SF, Hu ZD, Li J, Yu YS, Lin J, Shan B, Du Y, Han Y, Guo S, Wei LH, Wu L, Zhang H, Kong J, Hu YJ, Ai XM, Zhuo C, Su DH, Yang Q, Jia B, Huang W (2016). Resistance trends among clinical isolates in China reported from CHINET surveillance of bacterial resistance, 2005-2014. Clin Microbiol Infect.

[19] Chew KL, Lin RTP, Teo JWP (2017). *Klebsiella pneumoniae* in Singapore: Hypervirulent infections and the Carbapenemase threat. Front Cell Infect Microbiol.

[20] Kohler PP, Volling C, Green K, Uleryk EM, Shah PS, McGeer A (2017). Carbapenem resistance, initial antibiotic therapy, and mortality in *Klebsiella pneumoniae* bacteremia: a systematic review and meta-analysis. Infect Control Hosp Epidemiol.

[21] Javed H, Ejaz H, Zafar A, Rathore AW, Ikram ul H (2016). Metallo-beta-lactamase producing Escherichia coli and *Klebsiella pneumoniae*: a rising threat for hospitalized children. J Pak Med Assoc.

[22] Ma L, Wang JT, Wu TL, Siu LK, Chuang YC, Lin JC, Lu MC, Lu PL (2015). Emergence of OXA-48-producing *Klebsiella pneumoniae* in Taiwan. PLoS One.

[23] Liu Y, Feng Y, Wu W, Xie Y, Wang X, Zhang X, Chen X, Zong Z (2015). First report of OXA-181-producing *Escherichia coli* in China and characterization of the isolate using whole-genome sequencing. Antimicrob Agents Chemother.

[24] Yin D, Dong D, Li K, Zhang L, Liang J, Yang Y, Wu N, Bao Y, Wang C (2017). Hu F. clonal dissemination of OXA-232 Carbapenemase-producing *Klebsiella pneumoniae* in neonates. Antimicrob Agents Chemother.

[25] Tzouvelekis LS, Markogiannakis A, Piperaki E, Souli M, Daikos GL (2014). Treating infections caused by carbapenemase-producing *Enterobacteriaceae*. Clin Microbiol Infect.

[26] Qureshi ZA, Paterson DL, Potoski BA, Kilayko MC, Sandovsky G, Sordillo E, Polsky B, Adams-Haduch JM, Doi Y (2012). Treatment outcome of bacteremia due to KPC-producing *Klebsiella pneumoniae*: superiority of combination antimicrobial regimens. Antimicrob Agents Chemother.

[27] Zhang Y, Li P, Yin Y, Li F, Zhang Q (2017). In vitro activity of tigecycline in combination with rifampin, doripenem or ceftazidime against carbapenem-resistant *Klebsiella pneumoniae* bloodstream isolates. J Antibiot (Tokyo).

[28] Machuca I, Gutierrez-Gutierrez B, Gracia-Ahufinger I, Rivera Espinar F, Cano A, Guzman-Puche J, Perez-Nadales E, Natera C, Rodriguez M, Leon R, Caston JJ, Rodriguez-Lopez F, Rodriguez-Bano J, Torre-Cisneros J. Mortality associated with bacteremia due to Colistin-resistant *Klebsiella pneumoniae* with high-level Meropenem resistance: importance of combination therapy without Colistin and Carbapenems. Antimicrob Agents Chemother. 2017;61:e00406–17. 10.1128/AAC.00406-17 PMC5527651 28559247

[29] Hajjej Z, Gharsallah H, Naija H, Boutiba I, Labbene I, Ferjani M (2016). Successful treatment of a Carbapenem-resistant *Klebsiella pneumoniae* carrying Bla OXA-48 , Bla VIM-2 , Bla CMY-2 and Bla SHV- with high dose combination of imipenem and amikacin. IDCases.

[30] Albiero J, Sy SK, Mazucheli J, Caparroz-Assef SM, Costa BB, Alves JL, Gales AC, Tognim MC (2016). Pharmacodynamic evaluation of the potential clinical utility of Fosfomycin and Meropenem in combination therapy against KPC-2-producing *Klebsiella pneumoniae*. Antimicrob Agents Chemother.

[31] Poirel L, Kieffer N, Nordmann P (2016). In vitro evaluation of dual carbapenem combinations against carbapenemase-producing *Enterobacteriaceae*. J Antimicrob Chemother.

[32] Gugliandolo A, Caio C, Mezzatesta ML, Rifici C, Bramanti P, Stefani S, Mazzon E (2017). Successful ceftazidime-avibactam treatment of MDR-KPC-positive *Klebsiella pneumoniae* infection in a patient with traumatic brain injury: a case report. Medicine (Baltimore).

[33] Temkin E, Torre-Cisneros J, Beovic B, Benito N, Giannella M, Gilarranz R, Jeremiah C, Loeches B, Machuca I, Jimenez-Martin MJ, Martinez JA, Mora-Rillo M, Navas E, Osthoff M, Pozo JC, Ramos Ramos JC, Rodriguez M, Sanchez-Garcia M, Viale P, Wolff M, Carmeli Y. Ceftazidime-avibactam as salvage therapy for infections caused by Carbapenem-resistant organisms. Antimicrob Agents Chemother. 2017;61:e01964–16. 10.1128/AAC.01964-16 PMC5278727 27895014

[34] Holyk A, Belden V, Lee JJ, Musick W, Keul R, Britz GW, Lin J (2018). Ceftazidime/avibactam use for carbapenem-resistant *Klebsiella pneumoniae* meningitis: a case report. J Antimicrob Chemother.

[35] van Duin D, Lok JJ, Earley M, Cober E, Richter SS, Perez F, Salata RA, Kalayjian RC, Watkins RR, Doi Y, Kaye KS, Fowler VG, Jr., Paterson DL, Bonomo RA, Evans S, Antibacterial Resistance Leadership G. Colistin versus ceftazidime-avibactam in the treatment of infections due to Carbapenem-resistant *Enterobacteriaceae*. Clin Infect Dis. 2018;66:163–71. 10.1093/cid/cix783 PMC5850032 29020404

[36] Barnes MD, Winkler ML, Taracila MA, Page MG, Desarbre E, Kreiswirth BN, Shields RK, Nguyen MH, Clancy C, Spellberg B, Papp-Wallace KM, Bonomo RA. *Klebsiella pneumoniae* Carbapenemase-2 (KPC-2), substitutions at ambler position Asp179, and resistance to ceftazidime-avibactam: unique antibiotic-resistant phenotypes emerge from beta-lactamase protein engineering. MBio. 2017;8:e00528–17. 10.1128/mBio.00528-17 PMC5666153 29089425

[37] Bensman TJ, Wang J, Jayne J, Fukushima L, Rao AP, D'Argenio DZ, Beringer PM. Pharmacokinetic-Pharmacodynamic target attainment analyses to determine optimal dosing of ceftazidime-avibactam for the treatment of acute pulmonary exacerbations in patients with cystic fibrosis. Antimicrob Agents Chemother. 2017;61:e00988–17. 10.1128/AAC.00988-17 PMC5610479 28784670

[38] Davido B, Senard O, de Truchis P, Salomon J, Dinh A (2017). Monotherapy of ceftazidime-avibactam and ceftolozane-tazobactam: two effective antimicrobial agents against multidrug-resistant organisms except for NDM-1 isolates. Int J Infect Dis.

[39] Wenzler E, Deraedt MF, Harrington AT, Danizger LH (2017). Synergistic activity of ceftazidime-avibactam and aztreonam against serine and metallo-beta-lactamase-producing gram-negative pathogens. Diagn Microbiol Infect Dis.

[40] Davido B, Fellous L, Lawrence C, Maxime V, Rottman M, Dinh A. Ceftazidime-avibactam and Aztreonam, an interesting strategy to overcome beta-lactam resistance conferred by Metallo-beta-lactamases in *Enterobacteriaceae* and *Pseudomonas aeruginosa*. Antimicrob Agents Chemother. 2017;61:e01008–17. 10.1128/AAC.01008-17 PMC5571320 28630191

